# miRnalyze: an interactive database linking tool to unlock intuitive microRNA regulation of cell signaling pathways

**DOI:** 10.1093/database/bax015

**Published:** 2017-03-18

**Authors:** Sankha Subhra Das, Mithun James, Sandip Paul, Nishant Chakravorty

**Affiliations:** 1 School of Medical Science and Technology, Indian Institute of Technology, Kharagpur, West Bengal 721302, India; 2 National Brain Research Centre, Manesar, Haryana 122051, India

## Abstract

The various pathophysiological processes occurring in living systems are known to be orchestrated by delicate interplays and cross-talks between different genes and their regulators. Among the various regulators of genes, there is a class of small non-coding RNA molecules known as microRNAs. Although, the relative simplicity of miRNAs and their ability to modulate cellular processes make them attractive therapeutic candidates, their presence in large numbers make it challenging for experimental researchers to interpret the intricacies of the molecular processes they regulate. Most of the existing bioinformatic tools fail to address these challenges. Here, we present a new web resource ‘miRnalyze’ that has been specifically designed to directly identify the putative regulation of cell signaling pathways by miRNAs. The tool integrates miRNA-target predictions with signaling cascade members by utilizing TargetScanHuman 7.1 miRNA-target prediction tool and the KEGG pathway database, and thus provides researchers with in-depth insights into modulation of signal transduction pathways by miRNAs. miRnalyze is capable of identifying common miRNAs targeting more than one gene in the same signaling pathway—a feature that further increases the probability of modulating the pathway and downstream reactions when using miRNA modulators. Additionally, miRnalyze can sort miRNAs according to the seed-match types and TargetScan Context ++ score, thus providing a hierarchical list of most valuable miRNAs. Furthermore, in order to provide users with comprehensive information regarding miRNAs, genes and pathways, miRnalyze also links to expression data of miRNAs (miRmine) and genes (TiGER) and proteome abundance (PaxDb) data. To validate the capability of the tool, we have documented the correlation of miRnalyze’s prediction with experimental confirmation studies.

**Database URL:** http://www.mirnalyze.in

## Introduction

Signal transduction pathways are known to be the fundamental regulators of cellular development and pathophysiological processes. The progressive demands of survival, growth and development in living systems require achieving different biological outcomes. Such milestones are in turn manifestations of the differential regulation of various cellular signaling cascades. These modulations in expression of cellular signaling pathway members (genes and proteins) are mediated by various genetic and epigenetic mechanisms. Small non-coding RNA molecules known as microRNAs (miRNAs) are beginning to gain importance as one of the robust instruments for regulating gene expression patterns, and hence cellular signaling cascades (1). miRNAs are 22–25 nucleotide long non-coding RNA molecules which bind to target sites on mRNAs having partial or total complementarity (2). The human genome is believed to encode for nearly 30 000 genes. In contrast to this, only around 1000 miRNAs have been identified in human genome indicating that a single miRNA can implement its effect on several mRNAs (3, 4). Conversely, multiple miRNAs are known to target the same gene (5). Owing to their gene silencing ability, increased miRNA expression can possibly decrease the expression of target genes/proteins. Conversely, lower miRNA expression levels may result in up-regulation of gene expression. Considering these facts, it is possible that miRNAs play a vital role in the regulation of cellular signal transduction pathways. Experimental results from studies on various biological and pathophysiological processes provide evidence to this theory (6–9).

The mechanism of action of miRNAs [partial or total complementarity matching to 3′-untranslated regions (UTRs) of mRNAs] have led computational biologists to devise several target prediction tools like TargetScan, miRanda, PicTar, PITA, that provides us with the putative interactions of miRNAs with mRNAs, with each tool having its own advantages and disadvantages. Researchers have also attempted to analyse interactions among miRNAs, target genes and associated drugs using in-silico tools like PharmacomiR (10). Although curated lists of signaling pathway members are easily and freely available, researchers have limited access to tools that link miRNA target predictions to possible modulation of signaling pathways. DIANA-mirPath is an example of one such online web tool for enrichment analysis (11). miRGator is an internet-based database integrating several databases for target prediction, gene expression and genome annotation (12). Another freely available resource miTALOS analyzes tissue-specific regulation of signaling pathways by human and mouse microRNAs. Whilst these tools provide considerable information, they lack the crucial functionality of providing researchers choice of valuable target predictions for formulating experimental designs. Further, the existing tools do not provide users with a way to get the list of common miRNAs across different genes in the same or different cell signaling pathways.

The ever increasing number of miRNA-related publications evidence the fact that the interest in these non-coding RNA molecules and diseases is increasing rapidly. Researchers are being constantly challenged by the ever increasing pool of newly discovered miRNAs. miRbase is the most prominent database which systematically provides miRNA data to miRNA research community (13). Consequently, miRNA profiling experiments in diseased conditions, which are the backbone to identifying therapeutically relevant miRNAs, is becoming economically challenging. With the existing limitations in the pathway-miRNA resources available, scientists are left with the daunting task of experimentally sifting through enormous amounts of target-prediction data, which gravely handicaps their ability to identify and tease apart relevant molecular interactions. We have developed an online web based tool, ‘miRnalyze’ utilizing the Kyoto Encyclopedia of Genes and Genomes (KEGG) (14) database resource (dealing with genomes, biological pathways and diseases) and TargetScanHuman 7.1 (15) (a tool to predict miRNA targets). miRnalyze predicts putative miRNA targets in cellular signaling pathways directly through identifying involved genes in those pathways, and therefore integrates two of the most important molecular interactions, i.e. miRNA-gene and gene-gene. Additionally, it provides users with the ability to identify common miRNAs that have more than one target in the signal transduction pathways—a feature that is unique to miRnalyze. Further, the tool provides a sorted list of miRNAs based on their type of seed matches (16) and the TargetScan Context ++ score (15). The results thereby display the miRNAs in order of their relevance, highlighting the crucial miRNAs at the very top of the list. The above-mentioned features have the potential to help researchers identify and choose the most favorable miRNAs for expression profiling experiments. To demonstrate the competence of the tool, we have documented the correlation of miRnalyze’s prediction with experimental validation studies.

## Materials and methods

### Cell signaling pathway and gene relations

KEGG is an elaborated collection of information of genes/proteins involved in pathways across species (14, 17) that can be freely accessed through an application program interface (API). The KEGG database emphatically curates the members (genes/proteins) of the commonest and the most important signal transduction pathways in living systems, and hence was used for our purpose to extract information on genes involved in cell signaling pathways specifically for humans. Required information for our purpose was downloaded from KEGG server and stored in local system.

### miRNA target predictions for genes

**Figure 1:**
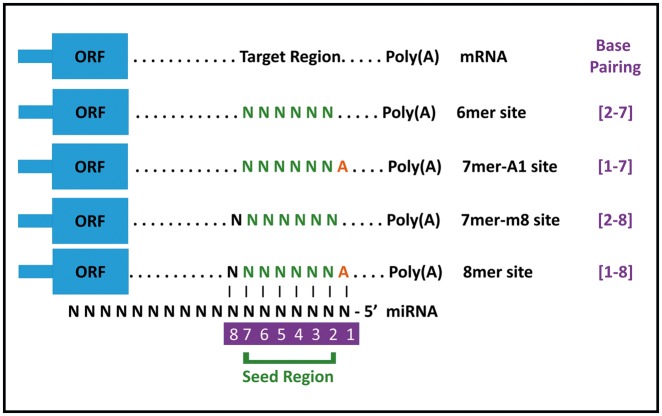
Different seed match regions of miRNAs. miRnalyze follows a hierarchical pattern (8mer > 7mer-m8 > 7mer-A1 > 6mer) for sorting miRNAs. ORF, Open Reading Frame.

MicroRNAs are believed to contain a ‘seed matching’ region that extends from second to seventh nucleotide (from 5′ end) of the miRNA. MicroRNA targets are predicted by the presence of complementary seed matching region(s) on the gene of interest. These seed match sites may include 6mer, 7mer-A1, 7mer-m8 and 8mer sites as described in Figure 1. Several bioinformatics-based target prediction resources are freely available on the internet. For the purpose of our analysis, we have specifically used the latest version of TargetScanHuman (Release 7.1) (15). TargetScan also provides a unique value known as ‘Context  ++ score’ against each miRNA, which is an index of the effectiveness of a given miRNA. This value is based on the seed match type, compensatory binding in the 3′ of the miRNA, AU content and position on the 3′-UTR. Lower values indicate stronger downregulation of expression.

### Expression data for microRNA and target and proteome abundance

The microRNA expression regulates the expression of specific genes in a signaling pathway. Hence, integration of both microRNA and target expression data is important for understanding overall regulation of signaling pathways. miRmine is a publicly available database which provides human microRNA expression data in a variety of human tissue. We have linked expression data for each microRNA in different human tissue by utilizing miRmine database (Guan Laboratory, University of Michigan). TiGER is another database for tissue specific gene expression and regulation. We have also linked expression data for each target member in various human tissue by using TiGER database (18). Additionally, we have linked proteome abundance data for each protein by using PaxDb database (19).

### miRnalyze designing

The frontend of the tool has been developed with Angular JS, a Model-View-Controller (MVC) JavaScript framework. The server side of the tool is developed with Laravel, a PHP framework. The Laravel backend acts as a RESTful web API that sends data to the frontend in JavaScript Object Notation (JSON) format. After its first load, the tool acts as a Single Page Application (SPA), which consumes data from the backend. The SPA behaviour allows for easy navigation between pages without reloading.

## Results

### miRnalyze web tool

**Figure 2:**
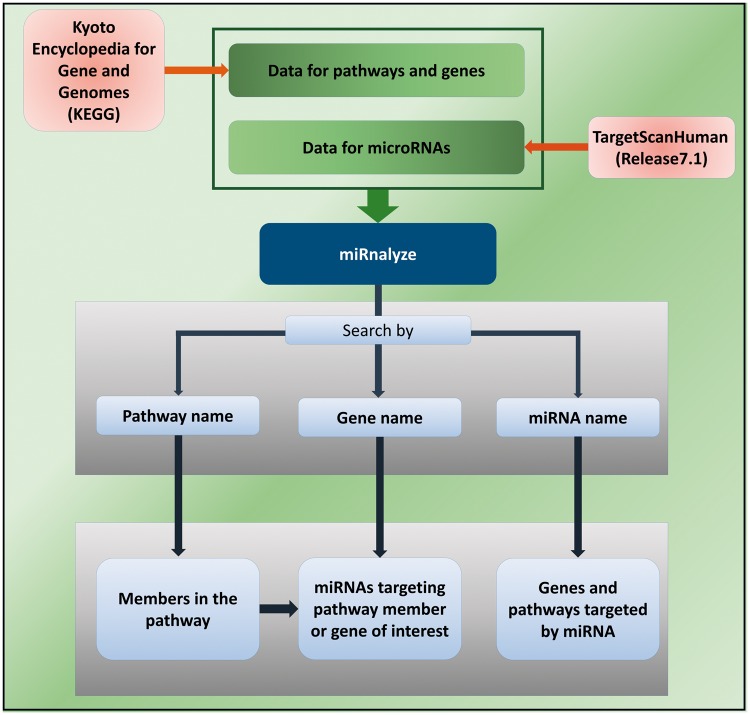
The schematic representation of miRnalyze.

miRnalyze is a dynamic web-based tool for enlisting miRNAs associated with particular cell signaling pathways and target genes. It utilizes two freely available massive database resources: Kyoto Encyclopedia of Genes and Genomes Database and TargetScanHuman (Release 7.1). The logistics based on which miRnalyze has been designed is shown schematically in Figure 2. In designing miRnalyze, we have endeavored to enhance the scope for researchers to identify predicted miRNAs involved cell signaling pathways by conjoining the information contained in these two databases. miRnalyze follows a hierarchical pattern of site efficacy as 8mer > 7mer-m8 > 7mer-A1 > 6mer (see Figure 1) while representing the data (20, 21). For each of the seed match group, miRnalyze tool arranges the miRNAs based on the Context ++ score. The most valuable miRNA is placed on top of the list. In order to reduce complexity, we have restricted the resource to function within the limits of human datasets. Furthermore, miRnalyze provides links to microRNA and target expression data and proteome abundance data to better understand overall regulation of signaling pathways.

### Workflow

**Figure 3:**
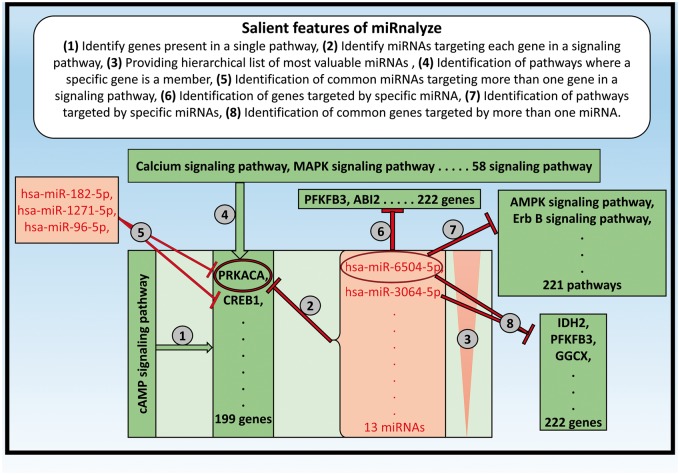
Salient features of miRnalyze along with specific example of each of the feature.

**Figure 4:**
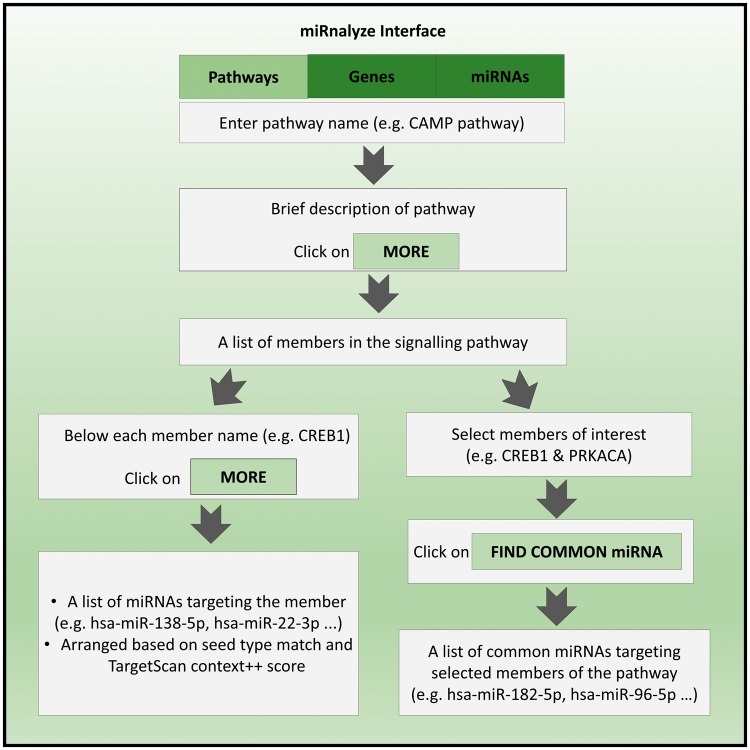
The miRnalyze workflow.

Salient features of miRnalyze along with specific example of each of the feature are represented in Figure 3. miRnalyze provides three separate search modules for the user: the pathways, the genes and the miRNAs, as depicted in Figure 4. All the three modules are interconnected and researchers can get details of the other modules after starting with one. miRnalyze’s custom-made sorting algorithms arrange enlisted miRNAs on the basis of seed matches and TargetScan Context ++ scores. A user-initiated search in the pathway module will lead to a list of genes involved in that particular pathway. Subsequently, a list of miRNAs that possibly target those genes individually can be obtained (Figures 3 and 4). Additionally, the common miRNAs targeting a set of genes can be obtained by pre-selecting the genes of interests (Figures 3 and 4). In the genes module, a list of targeted miRNAs and involved pathways can be obtained for a particular gene. A list of common target miRNAs for more than one gene can also be identified (Figure 3). The miRNA module allows users to identify the list of genes targeted by a particular miRNA. Further, the list of pathways downregulated by that miRNA can also be obtained (Figure 3).

### Prediction of miRnalyze

**Figure 5:**
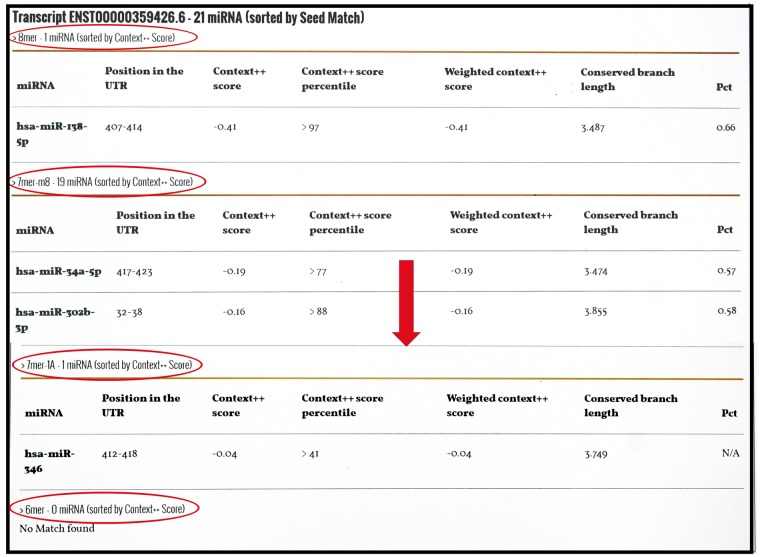
miRnalyze predicts miRNA based on seed match region as 8mer > 7mer-m8 > 7mer-A1 > 6mer (circle). For each of the seed match group, web tool arranges the miRNAs based on the Context ++ score (arrow).

miRnalyze can be used for experimental design and hypothesis construction. Results of miRnalyze are based on computational prediction only. It is obvious that computational prediction do not replace experimental confirmation but rather provide a weighty emphasis for such experiments. miRnalyze predicts microRNA targets in cellular signaling pathways directly through identifying involved genes in those pathways. We showed that miRnalyze prediction includes experimentally confirmed dysregulated miRNAs which cause disease through altering the pathway (Table 1). miRnalyze predicted list of miRNAs are sorted based on seed match sites and TargetScan context ++ score. The sorting is exemplified in Figure 5. Prediction of miRNAs targeting different cell signaling pathways enhance the understanding of miRNA-pathway relationship as well as serve as basis for development of biomarkers in disease diagnosis and therapies. miRnalyze also predicts miRNA that targets more than one genes. Using miRnalyze, we predicted miRNA targeting more than one overexpressed genes in different types of cancer and compared the miRnalyze prediction with miRNA confirmed by previous experimental study (Table 2).

**Table 1:** miRnalyze prediction includes experimentally confirmed dysregulated miRNAs in altered pathway

Disease	Altered Pathway	Experimentally confirmed dysregulated miRNAs	miRnalyze prediction includes
Chronic myelocytic leukemia	PI3K-AKT	hsa-miR-181c (22)	Yes
Cervical cancer	PTEN/AKT/FOXO1	hsa-miR-181a (23)	Yes
Hepatocellular carcinoma	AKT	hsa-miR-222 (24)	Yes
Ischemia-reperfusion	Apoptosis	hsa-miR-613 (25)	Yes

**Table 2:** Example of miRNA targeting more than one overexpressed genes predicted by miRnalyze and compare the prediction with previous experimental study

Disease	Overexpressed genes	miRNA targeting more than one genes predicted by miRnalyze	miRNA targeting more than one genes showing by previous experimental study	
			miRNA	Reference
Pancreatic cancer	CCND1, NT5E, PLAU, STMN1, YWHAZ	hsa-miR-193a-3p, hsa-miR-193b-3p	hsa-miR-193b	(26)
Glioblastoma	PDGFA, IGF2R, STAT3	hsa-miR-506-3p, hsa-miR-6835-3p, hsa-miR-124-3p.2	hsa-miR-506	(27–29)
Hepatocellular carcinoma	BMF, DDIT4, TIMP3	hsa-miR-181-5p, hsa-miR-221-3p, hsa-miR-222-3p, hsa-miR-4262	hsa-miR-221hsa-miR-222	(30–32)

## Discussion

Recent empirical evidence from across the globe strongly emphasize the implications of differential miRNA expression patterns in health and disease. Dysregulated miRNA expression patterns are known to be associated with development of several diseases (33). These small RNAs molecules are quite easy to target therapeutically and experimentally as they have short sequences and are conserved among many vertebrates (34). Another property that makes miRNAs interesting is their ability to target several genes in cellular signaling pathways. By targeting disease-linked miRNAs, modulation of entire signaling pathway may be possible. With the development of synthetic miRNA modulators like miRNA-mimics and antagomirs, the prospects of using miRNA modulators as therapeutic candidates is fast gaining recognition. Moreover, miRNA modulators have significant advantages over small molecule- and protein-based drug therapeutics. Major challenges associated with small drug- and protein-based drug molecules are slow lead optimization, variable selectivity and potency, and ability to target only certain classes of proteins (35). In contrast, miRNAs have rapid lead optimization, high selectivity and potency and ability to target virtually all genes (36).

With rapid progress of molecular biology technologies, more than thousands of miRNAs have been identified (4). This large number of miRNAs makes experimental studies more complex and time consuming. The one-to-many and many-to-one relationship between miRNAs and genes makes it difficult for researchers to identify the plausible miRNAs that may be targeted for modulate signaling pathways, and hence to delineate the biological processes under investigation. A deeper understanding of these delicate molecular interaction demands a reductionist in silico approach prior to experimental validation of probabilistic predictions. The web resource—miRnalyze has been designed to provide users with a heuristic tool to identify the key miRNA-cellular signaling cascade interactions. Initially, miRnalyze arranges the enlisted miRNAs based on hierarchical pattern of site efficacy as 8mer > 7mer-m8 > 7mer-A1 > 6mer. This is followed by sorting of miRNAs of each seed match group based on unique TargetScan Context ++ score value. The above feature helps researchers to identify the most relevant miRNAs for expression profiling while planning for experiments. miRnalyze includes novel feature to get common miRNAs which target more than one gene simultaneously.

Thus, miRnalyze is designed to identify the interaction among miRNAs, genes and cell signaling pathways integrating KEGG pathway database and TargetScanHuman 7.1 target prediction tool. KEGG is one of the earliest pathway databases developed (37) repositing well-structured and most appropriate information regarding biological pathways. Being one of the most exhaustive pathway databases available and surpassing most of the other contemporary resources, KEGG appears to serve as the most formidable backbone for our purpose. Among the various target prediction tools, we have specifically selected TargetScan (version TargetScanHuman 7.1) owing to its high degree of precision (∼50%) (38). Furthermore, the final score calculated by TargetScan is known to correlate with protein downregulation (39). It includes many parameters when describing score, such as the seed match type, compensatory binding in the 3′ of the miRNA, AU content and position on the 3′-UTR, which enable identification of the most relevant miRNAs. Moreover, TargetScan is updated regularly (3, 16, 20) adding to its credibility.

The interface of this web tool is very simple to use and produces nested data for easy interpretation. Therefore, miRnalyze is a useful tool for developing hypotheses in miRNA-mediated cell signaling pathways. We believe that miRnalyze has immense value for aiding researchers in predicting miRNA-related regulation of cell signaling pathway in the future.

### Future work

In the future, we will expand the miRnalyze by integrating more data from freely available resources such as KEGG pathway database and TargetScan target prediction tool.

## Funding

This work has been supported by the Institute Scheme for Innovative Research and Development (ISIRD), funded by Sponsored Research & Industrial Consultancy (SRIC), Indian Institute of Technology, Kharagpur, India [grant number IIT/SRIC/SMST/BHA/2015-16/122]. S.S.D. is supported by a fellowship funded by Council of Scientific and Industrial Research (CSIR), India [CSIR sanction number 09/081(1267)/2015-EMR-I].

*Conflict of interest*. None declared.
